# Care Next Door: Community Outpatient Esketamine Across Urban and Rural Clinics

**DOI:** 10.1192/j.eurpsy.2026.10607

**Published:** 2026-07-31

**Authors:** J. García Jiménez, S. Posik, B. Herrejón, L. Gutiérrez-Rojas

**Affiliations:** Psychiatry, Hospital Universitario Clínico San Cecilio, Andalusian Health Service (SAS), Granada, Spain

## Abstract

**Introduction:**

Treatment-resistant depression (TRD) is highly burdensome. Intranasal esketamine is effective, yet hospital-centric delivery adds travel and continuity barriers that may hinder adherence and outcomes. We hypothesize that **community outpatient, patient-proximal delivery**—often with the patient’s usual psychiatrist—improves effectiveness, engagement and acceptability, consistent with Spanish implementation experience and expert consensus (Molero et al. XLI Congr Nac Enferm Salud Ment 2024; — —; Ramos-Quiroga et al. Eur J Psychiatry 2025; 39(3) 100313; Young et al. Neurosci Appl 2024; 3 105240).

**Objectives:**

Primary: evaluate the effectiveness of intranasal esketamine for TRD in community outpatient clinics.

Secondary: trajectories of functioning, suicidality, well-being and treatment satisfaction; engagement/continuity and tolerability; exploratory association between proximity/continuity and outcomes.

**Methods:**

Multicentre, prospective, pragmatic cohort across urban and rural public community mental-health clinics; consecutive recruitment. Adults with TRD (≥2 adequate antidepressant failures) initiated esketamine per routine care. Assessments: baseline; weeks 2, 4, 8, 16, 24; program discharge; and 3/6-month post-discharge. Outcomes: **MADRS** (primary; response ≥50%; remission ≤10), **SDS**, **C-SSRS **, **SHS**, **SWLS**, **TSQM-9**; engagement (retention, attendance, continuity with the same psychiatrist); safety/tolerability. Planned analyses: mixed-effects models and time-to-event.

**Results:**

Preliminary subset (n = 7, single site); median age **55** years. **MADRS** means: **44.1** (baseline) → **35.1** (wk-2) → **32.3** (wk-4) → **26.4** (wk-8) → **24.4** (wk-12); **last-observation mean 19.0**; mean absolute change **−25.1** (≈**−56.6%**). **Response** 4/7 (57.1%); **remission** 2/7 (28.6%). **SDS** mean: **28.1** (baseline) → **16.0** (last); mean change **−12.1**. **C-SSRS** median: **4** (baseline) → **0** (last). Mean exposure **4.9 months**; at cut-off: **5/7** in treatment, **1/7** suspended for lack of efficacy, **1/7** discharged with **4-month** post-discharge stability. No discontinuations for safety recorded; adverse-event monitoring ongoing.

**Conclusions:**

Early real-world signals indicate that **near-patient outpatient delivery** yields rapid, clinically meaningful improvements in symptoms, functioning and suicidality with good feasibility and retention. Findings support the hypothesis that **proximity and relational continuity** act as **outcome amplifiers** in TRD, aligning with Spanish implementation experience and expert recommendations on practical delivery and adequate duration (Molero et al. XLI Congr Nac Enferm Salud Ment 2024; — —; Ramos-Quiroga et al. Eur J Psychiatry 2025; 39(3) 100313; Young et al. Neurosci Appl 2024; 3 105240).

**Disclosure of Interest:**

None Declared

